# A Novel DNA Replication-Related Signature Predicting Recurrence After R0 Resection of Pancreatic Ductal Adenocarcinoma: Prognostic Value and Clinical Implications

**DOI:** 10.3389/fcell.2021.619549

**Published:** 2021-03-04

**Authors:** Zengyu Feng, Kexian Li, Jianyao Lou, Mindi Ma, Yulian Wu, Chenghong Peng

**Affiliations:** ^1^Department of General Surgery, Pancreatic Disease Center, Research Institute of Pancreatic Diseases, Ruijin Hospital, Shanghai Jiao Tong University School of Medicine, Shanghai, China; ^2^State Key Laboratory of Oncogenes and Related Genes, Institute of Translational Medicine, Shanghai Jiao Tong University, Shanghai, China; ^3^Department of General Surgery, The Second Affiliated Hospital, School of Medicine, Zhejiang University, Hangzhou, China; ^4^Department of Nuclear Medicine, The Second Affiliated Hospital, School of Medicine, Zhejiang University, Hangzhou, China

**Keywords:** DNA replication, pancreatic ductal adenocarcinoma, prognostic signature, risk score, R0 resection, recurrence

## Abstract

The aim of any surgical resection for pancreatic ductal adenocarcinoma (PDAC) is to achieve tumor-free margins (R0). R0 margins give rise to better outcomes than do positive margins (R1). Nevertheless, postoperative morbidity after R0 resection remains high and prognostic gene signature predicting recurrence risk of patients in this subgroup is blank. Our study aimed to develop a DNA replication-related gene signature to stratify the R0-treated PDAC patients with various recurrence risks. We conducted Cox regression analysis and the LASSO algorithm on 273 DNA replication-related genes and eventually constructed a 7-gene signature. The predictive capability and clinical feasibility of this risk model were assessed in both training and external validation sets. Pathway enrichment analysis showed that the signature was closely related to cell cycle, DNA replication, and DNA repair. These findings may shed light on the identification of novel biomarkers and therapeutic targets for PDAC.

## Introduction

Pancreatic ductal adenocarcinoma (PDAC) accounts for over 90% cases of pancreatic malignancies, and is characterized by an overall 5 years survival rate of less than 9% (Hackeng et al., 2016; Siegel et al., 2019). Over the past decade, its incidence has been increasing in many countries, making PDAC a major global health challenge (Kamisawa et al., 2016). Surgery is currently the only therapeutic strategy that potentially cures this devastating disease (Kamisawa et al., 2016). Complete resection with no microscopic residual disease (R0 resection) is considered the main objective of surgery (Bockhorn et al., 2014; Baldwin et al., 2016). R0 margins give rise to significantly longer recurrence-free survival (RFS) compared with R1 margins (positive resection margins). However, even if R0 resection is achieved, more than half of patients subsequently experience local recurrence or distant metastases within 2 years of surgery (Kim et al., 2017; Strobel et al., 2017; Ghaneh et al., 2019; Tummers et al., 2019). Clinical features including but not limited to tumor size and tumor grade have been associated with tumor recurrence (Abe et al., 2020; Kim et al., 2020; Yoon et al., 2020); nevertheless, their predictive ability was not always satisfactory in R0-treated patients. Effective prediction of recurrence may help to tailor adjuvant chemotherapy and postoperative surveillance in this subgroup of patients.

DNA replication represents an under-explored source of prognostic markers that could be employed to predict tumor recurrence (Trenner and Sartori, 2019). The standard regimens after surgery, radiotherapy and chemotherapy, both kill cancer cells by amplifying genomic instability and by ultimately activating cell death pathways (Slade, 2020). Faithful execution of the DNA replication program through accurate replication and timely repair of damage to DNA is essential for cancer cell propagation and genomic stability, contributing to chemoresistance and radioresistance (Pilié et al., 2019; Kanellou et al., 2020; Wengner et al., 2020). Because of the cardinal importance of DNA replication in cancer, approaches targeting this biological process may provide a hopeful adjuvant therapy to improve PDAC prognosis. For example, poly ADP-ribose polymerase (PARP) inhibitors, which induce cell death via replication stress-induced mitotic catastrophe, have demonstrated anti-tumor efficacy in several clinical trials (Zhu et al., 2020). For these reasons, studying the role of DNA replication-related genes in cancer prognosis may assist individualized patient management.

Therefore, in the present study, we analyzed R0-resected PDAC patients using the MTAB-6134 and The Cancer Genome Atlas (TCGA) datasets. We measured the ability of expression of DNA replication-related genes to predict recurrence, and constructed a 7-gene risk model with powerful predictive ability in both training and validation datasets. This signature is closely associated with cell cycle and DNA repair, two key biological processes that are involved in response to chemotherapy and radiotherapy. These findings may lay the foundation for the development of individual treatment strategies and may potentially be implemented in clinical management.

## Materials and Methods

### Data Sources

The gene expression profiles and corresponding clinical information of the MTAB-6134 dataset were downloaded from the ArrayExpress database^1^. Normalized RNA-sequencing data and matched clinical data of TCGA dataset were retrieved from TCGA hub at UCSC Xena^2^. The MTAB-6134 dataset was adopted as the training set, and TCGA dataset was employed for external validation. Samples with follow-up time <1 month or lack of clinical information were excluded from further analyses. After careful review, 282 cases (234 cases with R0 resection and 48 cases with R1 resection) from MTAB-6134, and 124 cases (77 cases with R0 resection and 47 cases with R1 resection) from TCGA were analyzed in this study. No patients received neoadjuvant treatment in both cohorts. Clinical information regarding adjuvant chemotherapy was only provided in TCGA cohort. A total of 44 patients, who received adjuvant chemotherapy and had corresponding drug response information in TCGA cohort, were selected to investigate the association of the gene signature with a response to adjuvant treatment. All clinical data from included patients are detailed in Table 1. The DNA replication-related gene set (DNA_REPLICATION) was extracted from the MSigDB database^3^. Expression profiles of selected genes in tumor and normal samples were downloaded from the Ualcan website^4^.

**TABLE 1:** Clinicopathological characteristics of samples.

Parameter	Training set		Validation set	
	MTAB-6134 (*n* = 282)		TCGA-PAAD (*n* = 124)	
	R0 (*n* = 234)	R1 (*n* = 48)	R0 (*n* = 77)	R1 (*n* = 47)
**Gender**				
Female	104	14	37	21
Male	130	34	40	26
**Histological grade**				
G1	90	18	12	3
G2	106	22	42	31
G3	38	8	23	12
G4	NA	NA	NA	1
T stage				
T1	11	NA	4	NA
T2	33	6	10	3
T3	190	42	62	42
T4	NA	NA	1	2
**N stage**				
N0	69	3	23	9
N1	165	45	54	38

### PDAC Tissues

A total of sixty PDAC samples were harvested at the Department of General Surgery of Ruijin Hospital from April 2012 to August 2018. The follow-up lasted until February 2019. The dissected tissues were stored in liquid nitrogen. Written informed consent was obtained from all patients. This study was conducted in accordance with the Declaration of Helsinki, and the Ethics Committee of Ruijin Hospital affiliated with Shanghai Jiao Tong University approved the study.

### Construction of the DNA Replication-Related Gene Signature

In the MTAB-6134 dataset, DNA replication-related genes that were significantly associated with the RFS of R0 resected patients were identified using univariate Cox regression analysis and LASSO regression analysis. To avoid overfitting, a multivariate Cox regression analysis was further applied to determine an optimal risk signature with the minimum Akaike Information Criterion value. The risk score was computed as follows: Risk score = (coefficient ^∗^ expression value of gene 1) + (coefficient ^∗^ expression value of gene 2)+⋅+(coefficient ^∗^ expression value of gene X). The best cut-off value for next survival analysis was determined by X-tile software (Camp et al., 2004). Prognostic performance of this signature was evaluated by Kaplan–Meier (K-M) survival analysis, receiver operating characteristic (ROC) analysis, and calibration curves in both training and validation cohorts.

### Nomogram Based on the Signature

Nomogram is a quantitative method that is widely used for individual patient survival prediction. Survival-associated factors derived from the Univariate Cox regression analysis in MTAB-6134 cohort were identified. Based on the 234 patients in MTAB-6134 cohort, a nomogram comprised prognostic indicators was established to predict the 1-, 2-, and 3 years RFS by using the “rms” R package. Next, we used K-M survival analysis to assess the prognostic potential of this nomogram. In addition, we compared the predictive accuracy of this nomogram with that of clinical factors using the time-dependent area under the curve (AUC) generated from the “timeROC” R package.

### Functional and Pathway Analyses

Correlated genes of the risk score were screened using Spearman correlation analysis in MTAB-6134 dataset (| Spearman’s *r*| ≥ 0.3) and TCGA dataset (| Spearman’s *r*| ≥ 0.4). The correlated genes were then submitted to the Metascape database (Zhou et al., 2019) for function annotation and pathway enrichment.

### RNA Extraction and Quantitative Real-Time Polymerase Chain Reaction (qRT-PCR)

The total RNA from 60 PDAC samples (Ruijin cohort) was extracted using TRIzol reagent (Invitrogen, United States) and reverse-transcribed using an Evo M-MLV RT Kit (Accurate Biology, China). Real-time PCR was performed with an ABI 7900 instrument using ChamQ SYBR qPCR Master Mix (Vazyme, China). Quantitation was performed in triplicate. mRNA expression was calculated using the 2^–ΔΔ*CT*^ method and normalized to glyceraldehyde-3-phosphate dehydrogenase (GAPDH). The primers for the amplified mRNAs are summarized in Supplementary Table S1.

### Statistical Analysis

The statistical analyses and graphic production were conducted using R software (version 3.5.2). K-M survival curves with log-rank tests were generated using the “survival” package. ROC analyses were performed using the “survivalROC” package. Parameters in univariate and multivariate Cox analyses were derived from the “survival” package and were visualized using the “forestplot” package. The nomogram composed of independent prognostic indicators was established using the “rms” R package. Time-dependent area under the curve (AUC) values were calculated using the “timeROC” package. Boxplots depicting the distribution of gene expression and risk score were derived from the “ggpubr” package. *P* < 0.05 was considered significant.

## Results

### Survival Difference Between R0 and R1 Resected Patients

**FIGURE 1:**
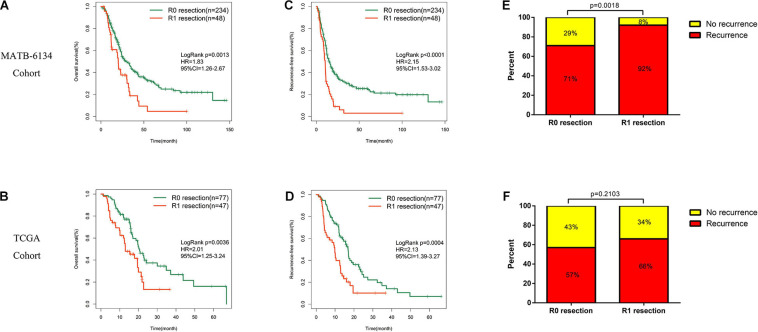
Survival difference between R0 and R1 treated patients. **(A,B)** K-M curves estimate OS difference between R0 and R1 margin. **(C,D)** K-M curves of RFS between patients with R0 and R1 resection. **(E,F)** Relationship of resection margin status and recurrence rate.

Survival analysis showed significantly decreased OS in the R1-resection group in the MTAB-6134 dataset (HR = 1.83, 95% CI = 1.26–2.67, *P* = 0.0013, Figure 1A) and TCGA dataset (HR = 2.01, 95% CI = 1.25–3.24, *P* = 0.0036, Figure 1B). Similarly, R1 resection was related to shorter RFS in the MTAB-6134 dataset (HR = 2.15, 95% CI = 1.53–3.02, *P* < 0.0001, Figure 1C) and TCGA dataset (HR = 2.13, 1.39–3.27, *P* = 0.0004, Figure 1D). PDAC patients with R0 resection had lower recurrence rates than did patients with R1 resection in the MTAB-6134 dataset (71 vs. 92%, *P* = 0.0018, Figure 1E) and TCGA dataset (57 vs. 66%, *P* = 0.2103, Figure 1F). These results suggest that patients with R0 resection had better survival and lower recurrence rates after surgery. Because R0 resection was the main objective of surgical resection, we endeavored to construct a recurrence prediction model for this group of PDAC patients.

### Development of the DNA Replication-Related Signature

**FIGURE 2:**
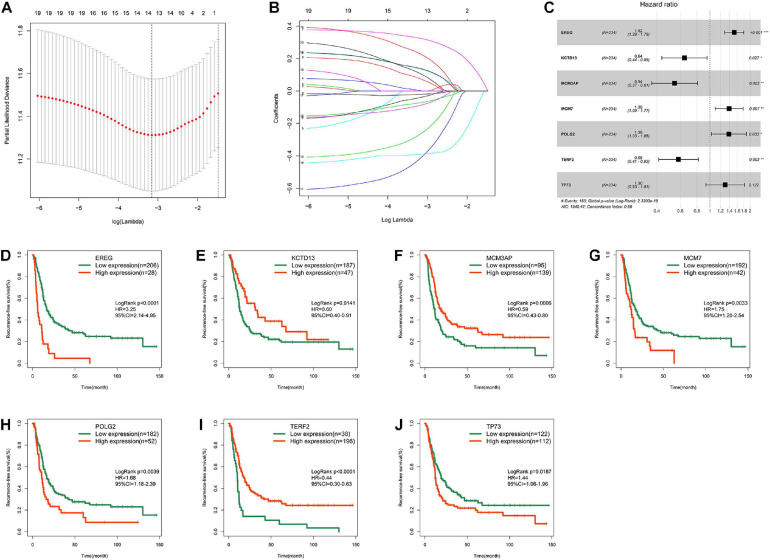
Development of the DNA replication-related signature. **(A)** Cross-validation for tuning parameter (lambda) screening in the LASSO regression model. **(B)** LASSO coefficient profiles of 20 prognostic DNA replication-related genes. **(C)** Forest plot of the seven DNA replication-related genes. **(D)** Survival cure for EREG. **(E)** Survival cure for KCTD13. **(F)** Survival cure for MCM3AP. **(G)** Survival cure for MCM7. **(H)** Survival cure for POLG2. **(I)** Survival cure for TERF2. **(J)** Survival cure for TP73. ^∗^*p* < 0.05; ^∗∗^*p* < 0.01; ^∗∗∗^*p* < 0.001.

Univariate Cox regression analysis identified 20 genes that were significantly associated with RFS in the MTAB-6134 dataset. The LASSO Cox regression algorithm was applied to these 20 genes to select the most useful predictive features, and 14 candidate genes were identified for further analysis (Figures 2A,B). Then, multivariate Cox regression analysis was applied and a risk prediction model comprising seven DNA replication-related genes was created finally (Figure 2C). According to the expression values and corresponding coefficients of the seven genes derived from multivariate Cox regression analysis, we established a risk-score formula: Risk score = 0.420 ^∗^ expression value of EREG −0.441 ^∗^ expression value of KCTD13 −0.610 ^∗^ expression value of MCM3AP + 0.330 ^∗^ expression value of MCM7 + 0.328 ^∗^ expression value of POLG2 −0.542 ^∗^ expression value of TERF2 + 0.263 ^∗^ expression value of TP73. Kaplan–Meier analysis showed that these seven individual genes adequately distinguished the RFS of patients in the MTAB-6134 cohort (Figures 2D–J). Expression profiles of the selected seven genes in normal and tumor samples are shown in Supplementary Figure S1.

Prognostic performance of risk signature in MTAB-6134 cohort.

**FIGURE 3:**
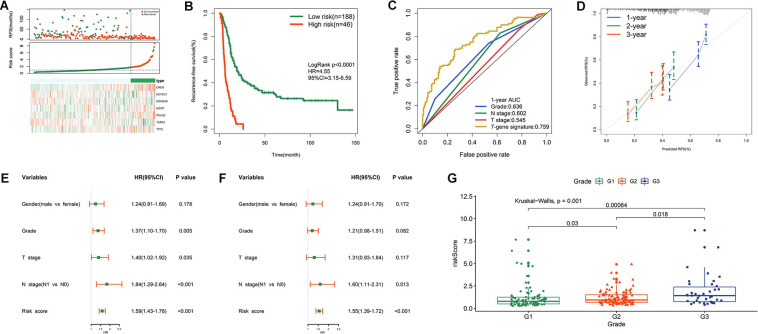
Prognostic performance of risk signature in MTAB-6134 cohort. **(A)** From top to bottom are the survival status distribution, risk score distribution and heat map analysis of seven genes. **(B)** RFS difference between low- and high-risk groups. **(C)** ROC analysis of the risk signature. **(D)** Calibration curves for risk score. **(E)** Univariate and **(F)** multivariate Cox regression analyses of clinical pathologic features and gene signature for RFS. **(G)** Distribution of risk scores in different histological grade.

Based on the optimal cut-off value determined by X-tile, we classified patients into high- and low- risk groups. The distribution of survival status, risk scores, and heatmap for expression levels of seven genes in training samples are shown in Figure 3A. The survival curve illustrated that patients in the low-risk group had significantly longer RFS (HR = 4.55, *P* < 0.0001) than did patients in the high-risk group (Figure 3B). ROC analysis showed that the risk score had moderate predictive value for short-term recurrence, with an AUC of 0.759 for 1 year RFS (Figure 3C). In addition, the 7-gene signature had a higher AUC than grade, N stage and T stage in predicting 1 year RFS (Figure 3C). The calibration curves indicated that the predicted survival probabilities using this model accorded well with the observed survival probabilities (Figure 3D). Univariate Cox regression analysis revealed that histological grade, T stage, N stage, and risk score were risk factors for RFS in MTAB-6134 cohort (Figure 3E). After further multivariate Cox regression of prognostic factors, risk score remained an independent predictor of patient survival (Figure 3F). Figure 3G illustrates that the risk score distributed differently with respect to the histological grade. We applied this risk signature in patients with R1 resection and found that the predictive accuracy was lower in this subgroup of patients with AUC value predicting 1 year RFS decreasing to 0.683 in the MTAB-6134 cohort (Supplementary Figure S2).

### Prognostic Performance of Risk Signature in TCGA Cohort

**FIGURE 4:**
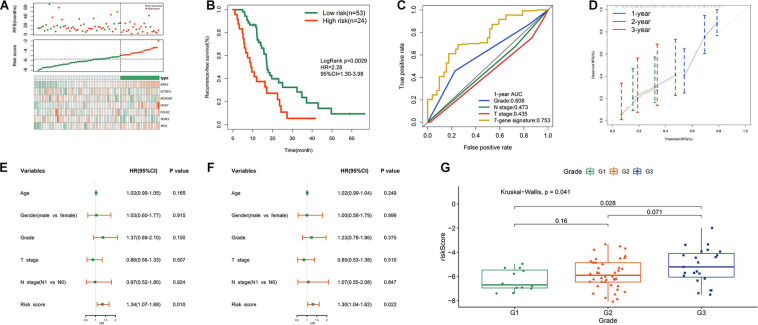
Prognostic performance of risk signature in TCGA cohort. **(A)** From top to bottom are the patients’ survival overview, risk score, and heat map of seven prognostic genes expression. **(B)** K–M curves evaluating the RFS between low- and high-risk groups. **(C)** ROC curve analysis of risk score in TCGA cohort. **(D)** Calibration curves for risk score. **(E)** Univariate and **(F)** multivariate Cox regression analyses of parameters for RFS. **(G)** Distribution of risk scores with respect to grade.

Figure 4A shows the distribution of the survival status, risk scores, and expression value of the seven genes in validation samples. K-M survival curves demonstrated that patients in the high-risk group had shorter survival times (Figure 4B). The risk score showed a satisfactory 1 year AUC of 0.757 in TCGA dataset (Figure 4C). The 7-gene signature exhibited better predictive performance for early recurrence than several traditional clinical indicators, including grade, N stage, and T stage (Figure 4C). The calibration plot revealed optimal agreement for prediction of 1-, 2-, and 3 years survival probability in TCGA cohort (Figure 4D). Both univariate and multivariate Cox analyses demonstrated the independent prognostic role of the risk signature for predicting tumor recurrence (Figures 4E,F). Figure 4G shows that higher histological grade correlated with higher risk score. However, the risk signature was no longer an appropriate tool for predicting early recurrence of R1-treated patients in TCGA cohort (Supplementary Figures S3A,B), indicating that this model has good specificity. Adjuvant chemotherapy is the standard treatment for PDAC after surgery. However, PDAC is refractory to the chemotherapeutic agents and currently no effective biomarkers are indicative of the response to adjuvant chemotherapy. We then investigated whether our model could stratify postoperative patients with different response to chemotherapeutic treatment in TCGA cohort. 44 patients who received adjuvant chemotherapy with related drug response information were divided into low- and high- risk group based on the medium value of risk score. Patients whose drug response is “clinical progressive disease” or “stable disease” were classified into chemotherapy-resistant group, while patients whose drug response is “complete response” or “partial response” were classified into chemotherapy-sensitive group. As shown in Supplementary Figure S3C, patients in low-risk group were more sensitive to adjuvant chemotherapy compared with patients in high-risk group. It could partly explain the lower recurrence rate of patients in low-risk group compared with high-risk group.

### Validation of the Prognostic Performance in a Local Cohort

**FIGURE 5:**
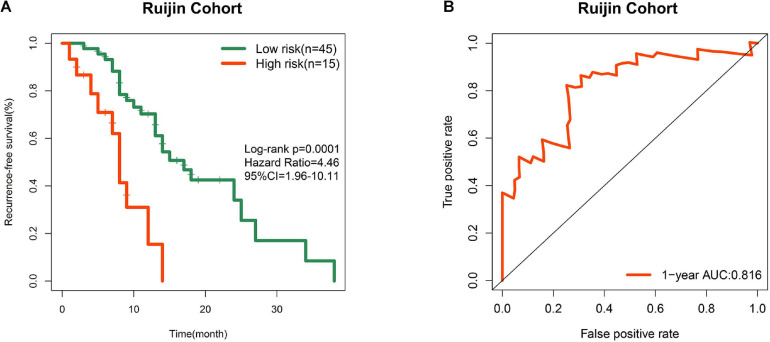
Prognostic validation in a local cohort. **(A)** K-M curve of RFS in Ruijin cohort. **(B)** ROC curve of the signature in predicting 1 year RFS in Ruijin cohort.

In order to improve the credibility of this signature, we subsequently validated the prognostic ability in the Ruijin cohort. K-M curves showed that the signature effectively captured the survival differences in RFS (Figure 5A). As illustrated in Figure 5B, the signature showed remarkable accuracy in predicting 1 year RFS as the AUC value was 0.816. These biological findings further confirmed the prognostic accuracy of the 7-gene signature.

### Comprehensive Analysis of EREG Expression in PDAC

**FIGURE 6:**
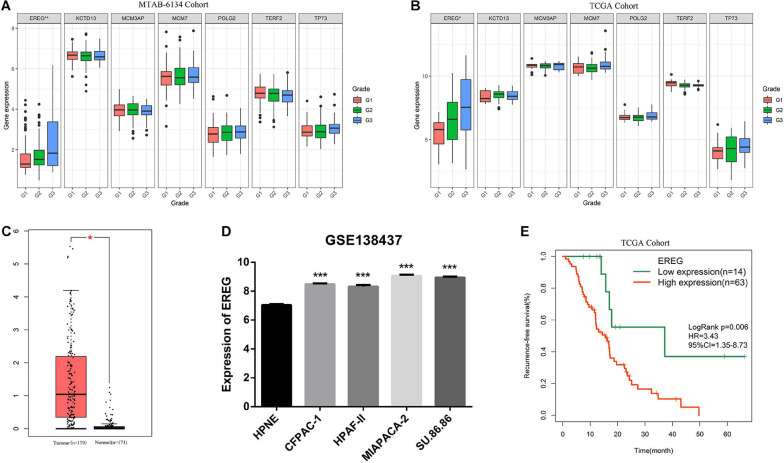
Comprehensive analysis of EREG expression in PDAC. **(A,B)** Expression of EREG in PDAC patients stratified by histological grade in MTAB-6134 and TCGA cohorts. **(C)** Expression of EREG in tumor tissues and normal tissues. **(D)** Expression of EREG in pancreatic cell lines based on the RNA-sequencing data downloaded from GSE138437 dataset. **(E)** Survival curve for EREG in TCGA cohort. *p < 0.05; **p < 0.01; ***p < 0.001.

To obtain an overview of the expression profiles of seven DNA replication-related genes in PDAC, we investigated the correlation between gene expression values and clinical features in both MTAB-6134 and TCGA datasets. It is worth noting that the expression of EREG was significantly higher in grade three patients than grades 2 or 1 patients in both MTAB-6134 and TCGA cohort (*P* < 0.05, Figures 6A,B). This result suggests that elevated expression levels of EREG might promote tumor malignancy. Expression levels of seven genes in patients categorized by T stage and N stage were also assessed in two cohorts. No common genes were distributed differently with respect. T stage and N stage (Supplementary Figure S4). The GEPIA database further confirmed the elevated expression levels of EREG in tumor tissues (Figure 6C). Except for tumor tissues, EREG was also found to be highly expressed in PDAC cell lines compared with HPNE cells, which are derived from normal human pancreatic duct (Figure 6D). In addition, elevated EREG expression levels were associated with shorter RFS in TCGA validation set (Figure 6E). These results jointly demonstrated that EREG may be a novel therapeutic target in PDAC treatment.

### Nomogram Construction

**FIGURE 7:**
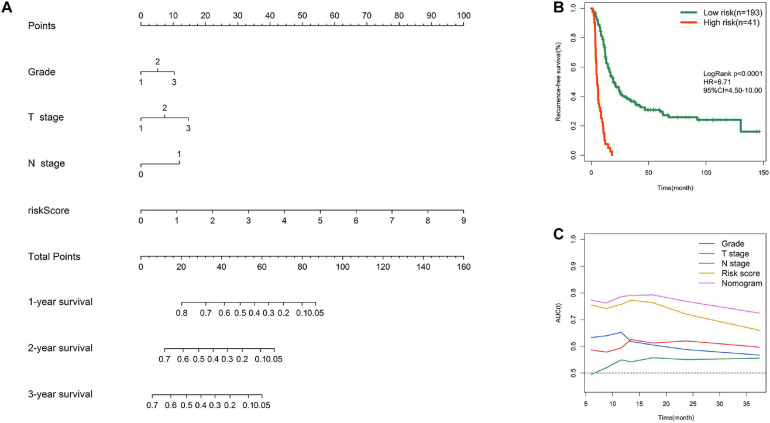
Nomogram construction. **(A)** Nomogram integrating risk score, grade, T stage, and N stage for RFS prediction. **(B)** K–M survival curve of nomogram in MTAB-6134 cohort. **(C)** Time-dependent AUC curves of prognostic indicators in MTAB-6134 cohort.

To facilitate clinical application, a graphic nomogram was developed based on the 234 patients in the MTAB-6134 cohort. Independent prognostic factors, including histological grade, T stage, N stage, and risk score, which were derived from the abovementioned univariate Cox analysis in MTAB-6134 cohort, were variables in the nomogram (Figure 7A). K-M analysis showed that this nomogram effectively discriminated patients with poor outcome from patients with favorable outcome (Figure 7B). The predictive efficacy of this nomogram was confirmed using AUC plots (Figure 7C). The risk score exhibited higher dynamic AUC value than grade, T stage, and N stage over time, suggesting that our risk model outperformed clinical indicators in terms of survival prediction.

### Functional Annotation and Pathway Enrichment

**FIGURE 8:**
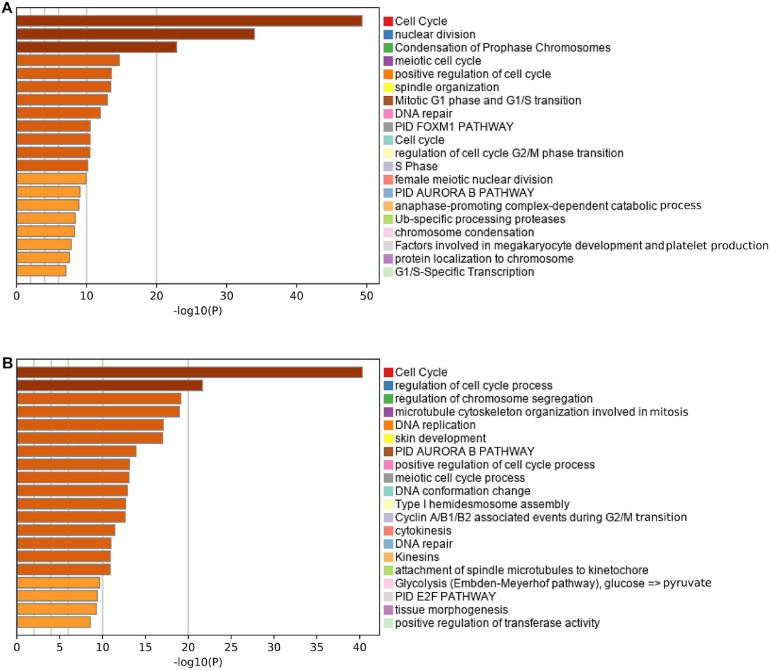
Functional annotation and pathway enrichment. **(A,B)** Enriched pathways of the genes positively correlated with risk score in MTAB-6134 cohort and TCGA cohort.

Pearson correlation analysis identified 193 and 621 genes that were co-expressed with the risk score in the MTAB-6134 and TCGA cohorts, respectively. Functional analysis revealed that 193 correlated genes in the MTAB-6134 cohort were involved in pathways related to cell cycle and DNA repair, suggesting an indispensable role in tumor recurrence (Figure 8A). Similar results were observed in TCGA cohort; 621 correlated genes in this cohort were found to be enriched in pathways associated with cell cycle, DNA replication, and DNA repair (Figure 8B).

## Discussion

Despite recent advances in the development of surgical techniques and perioperative management for patients with resectable PDAC, rates of postoperative morbidity, and mortality remain high (Ikuta et al., 2019). Unfortunately, due to the high degrees of heterogeneity, clinicopathological features including age, smoking history, TNM staging system, and histological grade provide limited information for estimating recurrence risk and outcome after surgery (Zhang et al., 2016). Considering the current situation of this devastating disease, any attempts help to predict tumor recurrence and guide the selection of reasonable treatment options after surgery should be welcomed. In this study, we systematically assessed the prognostic performance of DNA replication-related genes for predicting tumor recurrence and constructed a novel and robust signature including EREG, KCTD13, MCM3AP, MCM3, POLD2, TERF2, and TP73. The AUC values for 1 year RFS of this model were 0.759 and 0.757 in the MTAB-6134 and TCGA cohorts, respectively, indicating moderate predictive accuracy. Univariate and multivariate Cox analyses demonstrated that this signature was an independent and powerful predictor of short-term recurrence. Pathway enrichment analysis revealed that the signature was closely associated with DNA repair, cell cycle, and DNA replication.

Advancements in gene expression profiles derived from various high throughput technologies have improved our understanding of genetic and molecular alternations in PDAC. In recent years, many prognostic gene signatures have been developed by assessing crucial biological processes in PDAC progression, including autophagy (Yue et al., 2020), cell stemness (Feng et al., 2020) and immunological pathways (Wu et al., 2020). These models provided information for predicting OS. RFS prediction models are rare, to say nothing of risk models for the subgroup of R0 resected patients. In the present study, R0 margins were found to be associated with longer OS and RFS than were R1 margins in MTAB-6134 and TCGA cohorts. This finding is consistent with findings of earlier studies and indicates that survival analysis should be accurate for subgroup analysis. R0 resection is the aim of any curative resection, and the rate of R0 resection is considered a quality indicator for adequate oncological resection (Merkow et al., 2014). Thus, recurrence prediction for patients of this subgroup may have more clinical significance. Stratifying patients with resectable disease based on the predicted recurrence risk may facilitate personalized treatment and surveillance imaging. For patients with potentially resectable disease, if recurrence risk remains high even after complete resection, neoadjuvant therapy instead of upfront surgery is recommended to avoid futile surgery (Ikuta et al., 2019). For localized but unresectable disease, applying this model to biopsies obtained through a EUS-guided needle may help to tailor individualized treatments. That is to say, if the predicted recurrence risk is low, patients should be encouraged to receive neoadjuvant chemotherapy and potential complete resection. However, for localized but unresectable patients with high recurrence risk even after neoadjuvant chemotherapy and subsequent R0 resection, palliative treatments with the aim to improve life quality and ameliorate cancer complications may be more preferable.

Compelling evidence has revealed that DNA replication is closely associated with chemotherapy resistance, and several DNA replication-related genes have been confirmed as potential therapeutic targets in PDAC (Dunlop et al., 2020; Lloyd et al., 2020). Nevertheless, it remains unclear as to how DNA replication-related genes affect patient outcome, and this phenomenon has not been well reported. It would be significant to identify DNA replication-related biomarkers to predict recurrence risk and to explore novel targets of chemotherapy. In the present study, we systematically analyzed prognostic values of DNA replication-related genes for predicting tumor recurrence and integrated seven genes into a single signature using LASSO algorithm and multivariate Cox analysis. This signature effectively distinguished patients with significantly different postoperative survival in MTAB-6134 and TCGA cohorts. ROC analyses revealed that the signature had better predictive ability than clinical indicators, suggesting that the signature may serve as a complement to the current TNM staging system.

Among the seven genes, EREG, MCM7, and TP73 were previously reported to be involved in PDAC initiation and progression. EREG encodes a 46-amino acid protein that belongs to the epidermal growth factor receptor tyrosine kinase family (Toyoda et al., 1995). EREG is up-regulated in PDAC and stimulates pancreatic cancer cell growth *in vitro* (Zhu et al., 2000). MCM2-7 family members interact with one another to trigger the initial step of DNA replication (Evrin et al., 2009). High MCM7 expression results in poor outcomes in patients with PDAC (Peng et al., 2016; Liao et al., 2018). TP73 is activated in response to DNA damage and regulates many downstream biological processes including cell cycle and apoptosis (Barbhuiya et al., 2019). Ex2 + 4G > A genotypes of TP73 are significant predictors for tumor response, tumor resectability and overall survival in PDAC (Dong et al., 2009). TERF2 has remarkable tumor-specific effects; however, its role in PDAC remains unknown. TERF2 is a robust predictor of patient survival in cervical cancer (Benhamou et al., 2016) and oral carcinoma (Ozden et al., 2014). The role of TERF2 in cancers is primarily to promote angiogenesis (El Maï et al., 2014; Zizza et al., 2019). KCTD13, MCMAP3, and POLG2 have been rarely reported in published studies.

Despite our remarkable findings, this study has some limitations. First, detailed data of patient therapy were not available from the MTAB-6134 dataset. Although this signature effectively distinguished patients with various recurrence risks, individual survival benefits of chemotherapy and radiotherapy in each risk group remain unclear. Second, the current study was performed on retrospective data, and should therefore be tested using prospective data. Third, there are differences with respect to the definition of R0 resection: 0 mm tumor distance from resection margin in the United States and >1 mm in many centers in Europe and Australia (Demir et al., 2018). Although this model has been validated in both European (the MTAB-6134 cohort) and American populations (TCGA cohort), care should be taken to apply this model in patients of different nationalities. Fourth, more *in vivo* and *in vitro* experiments are needed to clarify biological function of the seven genes in terms of tumor progression.

In conclusion, we first reported a practical seven-gene signature based on the DNA replication-related genes and demonstrated that this signature could serve as a powerful predictor of tumor recurrence for patients with PDAC following R0 resection. The signature may help to provide reliable guidance and improved accuracy for treatment administration and surveillance imaging after surgery. However, the considerable variability with respect to the definition of R0 may impair accuracy and the predictive efficacy of this signature needs to be investigated in prospective studies.

## Data Availability Statement

The datasets presented in this study can be found in online repositories. The names of the repository/repositories and accession number(s) can be found in the article/Supplementary Material.

## Author Contributions

ZF: conceptualization, methodology, software, validation, formal analysis, and writing—original draft preparation. ZF and KL: investigation and data curation. ZF and JL: resources and visualization. CP and YW: writing—review and editing and supervision. CP: project administration and funding acquisition. All authors contributed to the article and approved the submitted version.

## Conflict of Interest

The authors declare that the research was conducted in the absence of any commercial or financial relationships that could be construed as a potential conflict of interest.

## Footnotes

1 https://www.ebi.ac.uk/arrayexpress/experiments/E-MTAB-6134/

2 https://tcga.xenahubs.net

3 https://www.gseamsigdb.org/gsea/msigdb/index.jsp

4 http://ualcan.path.uab.edu

## Abbreviations

AIC: Akaike Information Criterion
AUC: area under the curve
EGFR: epidermal growth factor receptor
K-M: Kaplan-Meier
PARP: Poly ADP-ribose polymerase
PDAC: pancreatic ductal adenocarcinoma
ROC: receiver operating characteristic
R0: tumor-free margin
R1: positive margin
TCGA: the Cancer Genome Atlas.

## References

[B1] Abe T. Amano H. Kobayashi T. Hattori M. Hanada K. Nakahara M. (2020). Efficacy of the physiobiological parameter-based grading system for predicting the long-term prognosis after curative surgery for resectable pancreatic cancer. *Eur. J. Surg. Oncol.* 47(3 Pt B) 613–619. 10.1016/j.ejso.2020.09.008 32978015

[B2] Baldwin S. Kukar M. Gabriel E. Attwood K. Wilkinson N. Hochwald S. N. (2016). Pancreatic cancer metastatic to a limited number of lymph nodes has no impact on outcome. *HPB (Oxford)* 18 523–528. 10.1016/j.hpb.2016.02.004 27317957 PMC4913131

[B3] Barbhuiya P. A. Uddin A. Chakraborty S. (2019). Compositional properties and codon usage of TP73 gene family. *Gene* 683 159–168. 10.1016/j.gene.2018.10.030 30316927

[B4] Benhamou Y. Picco V. Raybaud H. Sudaka A. Chamorey E. Brolih S. (2016). Telomeric repeat-binding factor 2: a marker for survival and anti-EGFR efficacy in oral carcinoma. *Oncotarget* 7 44236–44251. 10.18632/oncotarget.10005 27329590 PMC5190092

[B5] Bockhorn M. Uzunoglu F. G. Adham M. Imrie C. Milicevic M. Sandberg A. A. (2014). Borderline resectable pancreatic cancer: a consensus statement by the international study group of pancreatic surgery (ISGPS). *Surgery* 155 977–988. 24856119 10.1016/j.surg.2014.02.001

[B6] Camp R. L. Dolled-Filhart M. Rimm D. L. (2004). X-tile: a new bio-informatics tool for biomarker assessment and outcome-based cut-point optimization. *Clin. Cancer Res.* 10 7252–7259. 10.1158/1078-0432.ccr-04-0713 15534099

[B7] Demir I. E. Jäger C. Schlitter A. M. Konukiewitz B. Stecher L. Schorn S. (2018). R0 versus R1 resection matters after pancreaticoduodenectomy, and less after distal or total pancreatectomy for pancreatic cancer. *Ann. Surg.* 268 1058–1068. 10.1097/sla.0000000000002345 28692477

[B8] Dong X. Jiao L. Li Y. Evans D. B. Wang H. Hess K. R. (2009). Significant associations of mismatch repair gene polymorphisms with clinical outcome of pancreatic cancer. *J. Clin. Oncol.* 27 1592–1599. 10.1200/jco.2008.20.1111 19237629 PMC2668967

[B9] Dunlop C. R. Wallez Y. Johnson T. I. Bernaldo de Quirós Fernández S. Durant S. T. Cadogan E. B. (2020). Complete loss of ATM function augments replication catastrophe induced by ATR inhibition and gemcitabine in pancreatic cancer models. *Br. J. Cancer* 123 1424–1436. 10.1038/s41416-020-1016-2 32741974 PMC7591912

[B10] El Maï M. Wagner K. D. Michiels J. F. Ambrosetti D. Borderie A. Destree S. (2014). The telomeric protein TRF2 regulates angiogenesis by binding and activating the PDGFRβ promoter. *Cell Rep.* 9 1047–1060. 10.1016/j.celrep.2014.09.038 25437559

[B11] Evrin C. Clarke P. Zech J. Lurz R. Sun J. Uhle S. (2009). A double-hexameric MCM2-7 complex is loaded onto origin DNA during licensing of eukaryotic DNA replication. *Proc. Natl. Acad. Sci. U.S.A.* 106 20240–20245. 10.1073/pnas.0911500106 19910535 PMC2787165

[B12] Feng Z. Shi M. Li K. Ma Y. Jiang L. Chen H. (2020). Development and validation of a cancer stem cell-related signature for prognostic prediction in pancreatic ductal adenocarcinoma. *J. Transl. Med.* 18:360. 10.1186/s12967-020-02527-1 PMC7507616 32958051

[B13] Ghaneh P. Kleeff J. Halloran C. M. Raraty M. Jackson R. Melling J. (2019). The impact of positive resection margins on survival and recurrence following resection and adjuvant chemotherapy for pancreatic ductal adenocarcinoma. *Ann. Surg.* 269 520–529. 10.1097/sla.0000000000002557 29068800

[B14] Hackeng W. M. Hruban R. H. Offerhaus G. J. Brosens L. A. (2016). Surgical and molecular pathology of pancreatic neoplasms. *Diagn. Pathol.* 11:47. 10.1186/s13000-016-0497-z PMC4897815 27267993

[B15] Ikuta S. Sonoda T. Aihara T. Yamanaka N. (2019). A combination of platelet-to-lymphocyte ratio and carbohydrate antigen 19-9 predict early recurrence after resection of pancreatic ductal adenocarcinoma. *Ann. Transl. Med.* 7:461. 10.21037/atm.2019.08.35 31700897 PMC6803228

[B16] Kamisawa T. Wood L. D. Itoi T. Takaori K. (2016). Pancreatic cancer. *Lancet* 388 73–85. 26830752 10.1016/S0140-6736(16)00141-0

[B17] Kanellou A. Giakoumakis N. N. Panagopoulos A. Tsaniras S. C. Lygerou Z. (2020). The licensing factor Cdt1 links cell cycle progression to the DNA damage response. *Anticancer Res.* 40 2449–2456. 10.21873/anticanres.14214 32366388

[B18] Kim D. W. Lee S. S. Kim S. O. Kim J. H. Kim H. J. Byun J. H. (2020). Estimating recurrence after upfront surgery in patients with resectable pancreatic ductal adenocarcinoma by using pancreatic CT: development and validation of a risk score. *Radiology* 296 541–551. 10.1148/radiol.2020200281 32662759

[B19] Kim K. S. Kwon J. Kim K. Chie E. K. (2017). Impact of resection margin distance on survival of pancreatic cancer: a systematic review and meta-analysis. *Cancer Res. Treat.* 49 824–833. 10.4143/crt.2016.336 27561314 PMC5512376

[B20] Liao X. Han C. Wang X. Huang K. Yu T. Yang C. (2018). Prognostic value of minichromosome maintenance mRNA expression in early-stage pancreatic ductal adenocarcinoma patients after pancreaticoduodenectomy. *Cancer Manag. Res.* 10 3255–3271. 10.2147/cmar.s171293 30233242 PMC6130532

[B21] Lloyd R. L. Wijnhoven P. W. G. Ramos-Montoya A. Wilson Z. Illuzzi G. Falenta K. (2020). Combined PARP and ATR inhibition potentiates genome instability and cell death in ATM-deficient cancer cells. *Oncogene* 39 4869–4883. 10.1038/s41388-020-1328-y 32444694 PMC7299845

[B22] Merkow R. P. Bilimoria K. Y. Bentrem D. J. Pitt H. A. Winchester D. P. Posner M. C. (2014). National assessment of margin status as a quality indicator after pancreatic cancer surgery. *Ann. Surg. Oncol.* 21 1067–1074. 10.1245/s10434-013-3338-2 24337612

[B23] Ozden S. Tiber P. M. Ozgen Z. Ozyurt H. Serakinci N. Orun O. (2014). Expression of TRF2 and its prognostic relevance in advanced stage cervical cancer patients. *Biol. Res.* 47:61. 10.1186/0717-6287-47-61 25654471 PMC4335779

[B24] Peng Y. P. Zhu Y. Yin L. D. Zhang J. J. Guo S. Fu Y. (2016). The expression and prognostic roles of MCMs in pancreatic cancer. *PLoS One* 11:e0164150. 10.1371/journal.pone.0164150 27695057 PMC5047525

[B25] Pilié P. G. Tang C. Mills G. B. Yap T. A. (2019). State-of-the-art strategies for targeting the DNA damage response in cancer. *Nat. Rev. Clin. Oncol.* 16 81–104. 10.1038/s41571-018-0114-z 30356138 PMC8327299

[B26] Siegel R. L. Miller K. D. Jemal A. (2019). Cancer statistics 2019. *CA Cancer J. Clin.* 69 7–34. 30620402 10.3322/caac.21551

[B27] Slade D. (2020). PARP and PARG inhibitors in cancer treatment. *Genes Dev.* 34 360–394. 10.1101/gad.334516.119 32029455 PMC7050487

[B28] Strobel O. Hank T. Hinz U. Bergmann F. Schneider L. Springfeld C. (2017). Pancreatic cancer surgery: the new R-status counts. *Ann. Surg.* 265 565–573. 10.1097/sla.0000000000001731 27918310

[B29] Toyoda H. Komurasaki T. Uchida D. Takayama Y. Isobe T. Okuyama T. (1995). Epiregulin. A novel epidermal growth factor with mitogenic activity for rat primary hepatocytes. *J. Biol. Chem.* 270 7495–7500. 7706296 10.1074/jbc.270.13.7495

[B30] Trenner A. Sartori A. A. (2019). Harnessing DNA double-strand break repair for cancer treatment. *Front. Oncol.* 9:1388. 10.3389/fonc.2019.01388 r 31921645 PMC6921965

[B31] Tummers W. S. Groen J. V. Sibinga Mulder B. G. Farina-Sarasqueta A. Morreau J. Putter H. (2019). Impact of resection margin status on recurrence and survival in pancreatic cancer surgery. *Br. J. Surg.* 106 1055–1065. 30883699 10.1002/bjs.11115 PMC6617755

[B32] Wengner A. M. Scholz A. Haendler B. (2020). Targeting DNA damage response in prostate and breast cancer. *Int. J. Mol. Sci.* 21:8273. 10.3390/ijms21218273 PMC7663807 33158305

[B33] Wu G. Deng Z. Jin Z. Wang J. Xu B. Zeng J. (2020). Identification of prognostic immune-related genes in pancreatic adenocarcinoma and establishment of a prognostic nomogram: a bioinformatic study. *Biomed Res. Int.* 2020:1346045. 10.1155/2020/1346045 PMC7301181 32596278

[B34] Yoon S. J. Shin S. H. Yoon S. K. Jung J. H. You Y. Han I. W. (2020). Appraisal of 5-year recurrence-free survival after surgery in pancreatic ductal adenocarcinoma. *J. Hepatobiliary Pancreat. Sci.* 00 1–10. 10.1002/jhbp.815 32790012

[B35] Yue P. Zhu C. Gao Y. Li Y. Wang Q. Zhang K. (2020). Development of an autophagy-related signature in pancreatic adenocarcinoma. *Biomed. Pharmacother.* 126:110080. 10.1016/j.biopha.2020.110080 32203889

[B36] Zhang Q. Zeng L. Chen Y. Lian G. Qian C. Chen S. (2016). Pancreatic cancer epidemiology, detection, and management. *Gastroenterol. Res. Pract.* 2016:8962321. 10.1155/2016/8962321 PMC4749824 26941789

[B37] Zhou Y. Zhou B. Pache L. Chang M. Khodabakhshi A. H. Tanaseichuk O. (2019). Metascape provides a biologist-oriented resource for the analysis of systems-level datasets. *Nat. Commun.* 10:1523. 10.1038/s41467-019-09234-6 PMC6447622 30944313

[B38] Zhu H. Wei M. Xu J. Hua J. Liang C. Meng Q. (2020). PARP inhibitors in pancreatic cancer: molecular mechanisms and clinical applications. *Mol. Cancer* 19:49. 10.1186/s12943-020-01167-9 PMC7053129 32122376

[B39] Zhu Z. Kleeff J. Friess H. Wang L. Zimmermann A. Yarden Y. (2000). Epiregulin is up-regulated in pancreatic cancer and stimulates pancreatic cancer cell growth. *Biochem. Biophys. Res. Commun.* 273 1019–1024. 10891365 10.1006/bbrc.2000.3033

[B40] Zizza P. Dinami R. Porru M. Cingolani C. Salvati E. Rizzo A. (2019). TRF2 positively regulates SULF2 expression increasing VEGF-A release and activity in tumor microenvironment. *Nucleic Acids Res.* 47 3365–3382. 30698737 10.1093/nar/gkz041 PMC6468246

