# Supplementary material for: A Novel DNA Replication-Related Signature Predicting Recurrence After R0 Resection of Pancreatic Ductal Adenocarcinoma: Prognostic Value and Clinical Implications
Source: Front Cell Dev Biol. 2021 Mar 4;9:619549. doi: 10.3389/fcell.2021.619549 (PMC7969722; doi:10.3389/fcell.2021.619549)
Supplement: Supplementary file 1 [file Data_Sheet_1.PDF]

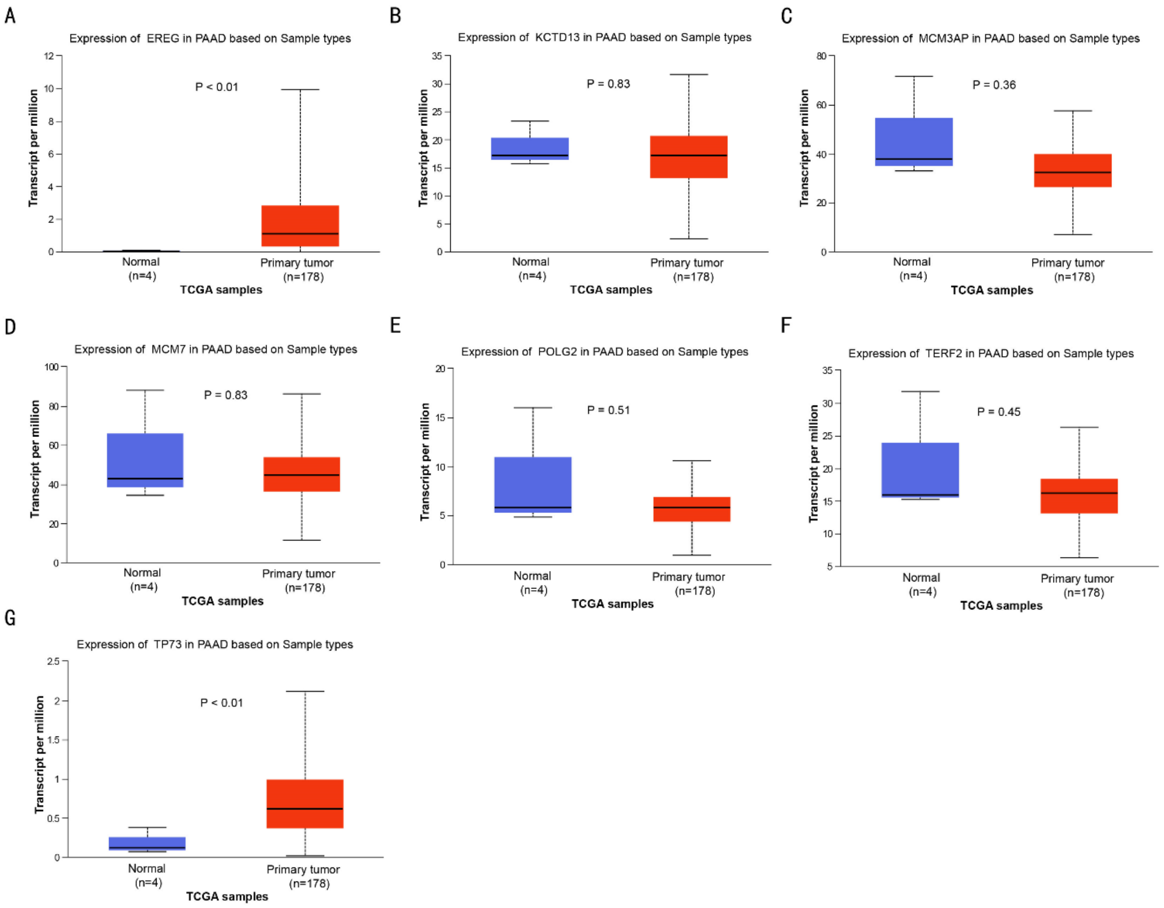

Supplementary Figure 1. Expression of seven genes in TCGA dataset.

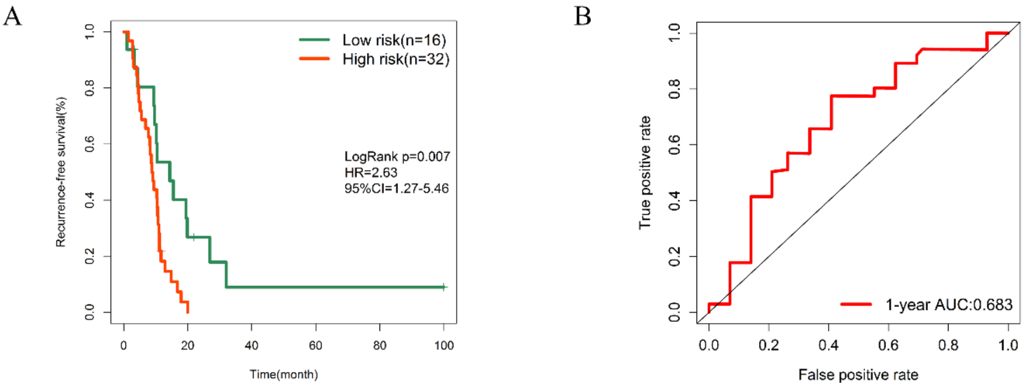

Supplementary Figure 2. Prognostic performance of the signature in R1 resected patients from MTAB-6134 dataset. (A) K-M survival curve. (B) ROC curve.

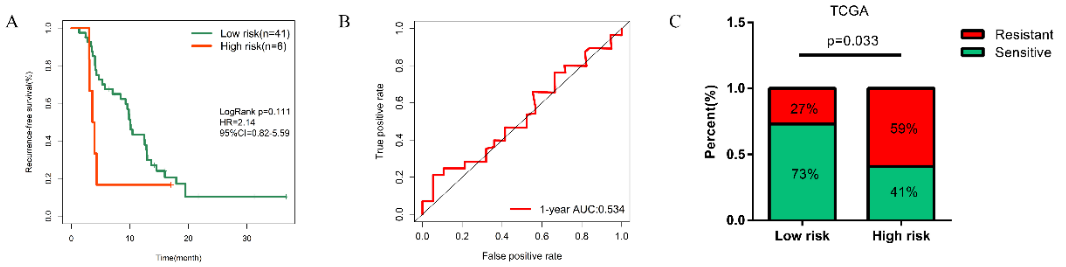

Supplementary Figure 3. Prognostic performance of the signature in R1 resected patients from TCGA dataset. (A) K-M survival curve. (B) ROC curve. (C) Correlation of risk score and the response to adjuvant chemotherapy in TCGA cohort.

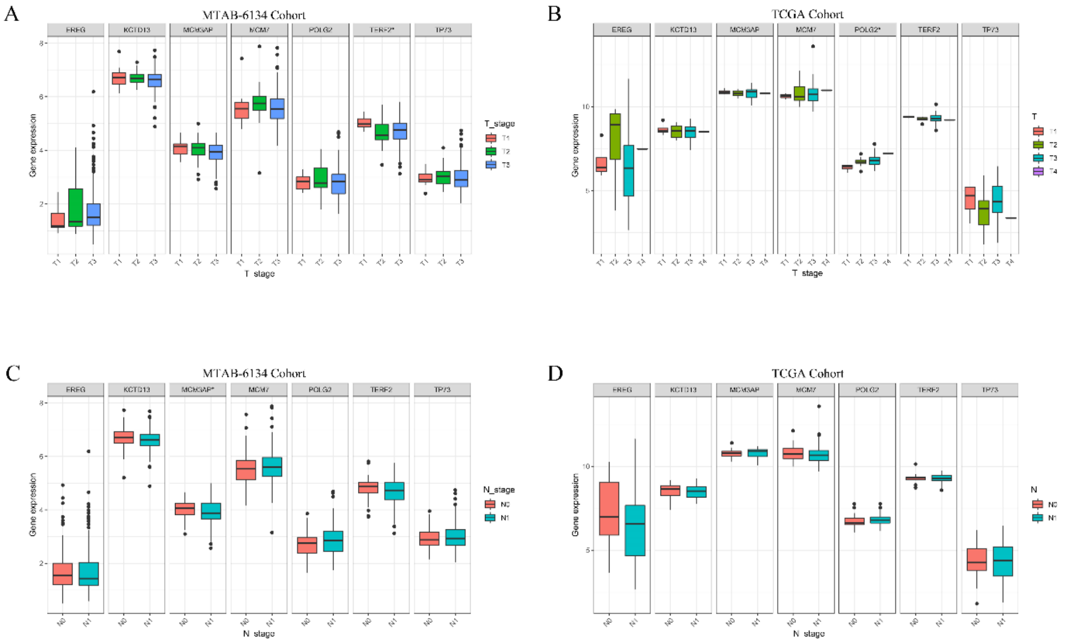

Supplementary Figure 4. Relationship of gene expression and clinical features. (A, B) Expression of seven genes in patients classified by T stage in MTAB-6134 and TCGA datasets. (C, D) Expression of seven genes in patients classified by N stage in MTAB-6134 and TCGA datasets.
