# Supplementary material for: A Novel DNA Replication-Related Signature Predicting Recurrence After R0 Resection of Pancreatic Ductal Adenocarcinoma: Prognostic Value and Clinical Implications
Source: Front Cell Dev Biol. 2021 Mar 4;9:619549. doi: 10.3389/fcell.2021.619549 (PMC7969722; doi:10.3389/fcell.2021.619549)
Supplement: Supplementary file 2 [file Table_1.DOCX]

Table. S1 Primers of amplified mRNAs.

| Primers | Sequence (5'-3') |
| --- | --- |
| GAPDH-F | GCACCGTCAAGGCTGAGAAC |
| GAPDH-R | TGGTGAAGACGCCAGTGGA |
| EREG-F | GTGATTCCATCATGTATCCCAGG |
| EREG-R | GCCATTCATGTCAGAGCTACACT |
| KCTD13-F | CCTGCACAACCGCAGTAACA |
| KCTD13-R | CCTGCCCGTAGAAAGACCAG |
| MCM3AP-F | GGGCGATCATCCTCCAGAC |
| MCM3AP-R | TCCTGTATCGTCCGACCAAATA |
| MCM7-F | GCCTGTGGGAAATATCCCTCG |
| MCM7-R | GTACCACCTGTCGGAACCC |
| POLG2-F | CCGGAGCTGTTGACGGAAA |
| POLG2-R | TTCCACTTAGGAAATGCCTTCTC |
| TERF2-F | GTACGGGGACTTCAGACAGAT |
| TERF2-R | CGCGACAGACACTGCATAAC |
| TP73-F | CGGGCCATGCCTGTTTACA |
| TP73-R | TGTCCTTCGTTGAAGTCCCTC |
