# Supplementary material for: A Novel DNA Replication-Related Signature Predicting Recurrence After R0 Resection of Pancreatic Ductal Adenocarcinoma: Prognostic Value and Clinical Implications
Source: Front Cell Dev Biol. 2021 Mar 4;9:619549. doi: 10.3389/fcell.2021.619549 (PMC7969722; doi:10.3389/fcell.2021.619549)
Supplement: Supplementary file 3 [file Table_2.DOCX]

Table. S2 Chemotherapeutic information of patients in TCGA cohort.

| sample | risk score | adjuvant treatment | drugs response |
| --- | --- | --- | --- |
| TCGA-XD-AAUG | -8.075076934 | Chemotherapy | Clinical Progressive Disease |
| TCGA-HZ-7918 | -7.494149023 | Chemotherapy | Complete Response |
| TCGA-F2-6880 | -7.394857015 | Chemotherapy | Complete Response |
| TCGA-YY-A8LH | -7.381631994 | Chemotherapy | Complete Response |
| TCGA-LB-A8F3 | -7.365894411 | Chemotherapy | Complete Response |
| TCGA-FB-AAPQ | -7.290207314 | Chemotherapy | Clinical Progressive Disease |
| TCGA-HZ-8637 | -7.147640051 | Chemotherapy | Complete Response |
| TCGA-F2-A44H | -7.094591071 | Chemotherapy | Complete Response |
| TCGA-3A-A9I5 | -6.994190131 | Chemotherapy | Complete Response |
| TCGA-Z5-AAPL | -6.930908532 | Chemotherapy | Complete Response |
| TCGA-IB-7645 | -6.91216703 | Chemotherapy | Complete Response |
| TCGA-IB-7891 | -6.806014973 | Chemotherapy | Complete Response |
| TCGA-IB-7888 | -6.722185909 | Chemotherapy | Stable Disease |
| TCGA-F2-7276 | -6.587442063 | Chemotherapy | Clinical Progressive Disease |
| TCGA-Q3-A5QY | -6.461868078 | Chemotherapy | Partial Response |
| TCGA-HZ-A49H | -6.442956173 | Chemotherapy | Complete Response |
| TCGA-FB-A78T | -6.437979575 | Chemotherapy | Complete Response |
| TCGA-RB-AA9M | -6.341995164 | Chemotherapy | Clinical Progressive Disease |
| TCGA-HZ-7923 | -6.280639103 | Chemotherapy | Complete Response |
| TCGA-3A-A9IX | -6.254259128 | Chemotherapy | Complete Response |
| TCGA-FB-AAQ6 | -6.216586593 | Chemotherapy | Complete Response |
| TCGA-FB-AAPP | -6.168677455 | Chemotherapy | Clinical Progressive Disease |
| TCGA-HZ-7920 | -6.125730894 | Chemotherapy | Complete Response |
| TCGA-US-A774 | -5.938296153 | Chemotherapy | Clinical Progressive Disease |
| TCGA-FB-AAPY | -5.790142031 | Chemotherapy | Complete Response |
| TCGA-3A-A9I9 | -5.729388725 | Chemotherapy | Stable Disease |
| TCGA-YB-A89D | -5.52052394 | Chemotherapy | Complete Response |
| TCGA-HZ-8001 | -5.293613397 | Chemotherapy | Complete Response |
| TCGA-IB-7889 | -5.293470388 | Chemotherapy | Clinical Progressive Disease |
| TCGA-S4-A8RM | -5.205193033 | Chemotherapy | Complete Response |
| TCGA-3A-A9IH | -4.986639507 | Chemotherapy | Stable Disease |
| TCGA-OE-A75W | -4.949234909 | Chemotherapy | Clinical Progressive Disease |
| TCGA-IB-A5SO | -4.861850096 | Chemotherapy | Clinical Progressive Disease |
| TCGA-3A-A9IC | -4.861364833 | Chemotherapy | Stable Disease |
| TCGA-LB-A9Q5 | -4.837493498 | Chemotherapy | Clinical Progressive Disease |
| TCGA-HZ-7924 | -4.746692667 | Chemotherapy | Complete Response |
| TCGA-IB-7651 | -4.50426982 | Chemotherapy | Clinical Progressive Disease |
| TCGA-2L-AAQE | -4.47056466 | Chemotherapy | Clinical Progressive Disease |
| TCGA-IB-A5SS | -4.402466764 | Chemotherapy | Partial Response |
| TCGA-3A-A9J0 | -4.315379136 | Chemotherapy | Clinical Progressive Disease |
| TCGA-FB-A5VM | -4.240983672 | Chemotherapy | Clinical Progressive Disease |
| TCGA-IB-7885 | -3.792489363 | Chemotherapy | Complete Response |
| TCGA-HZ-A77O | -3.339944842 | Chemotherapy | Clinical Progressive Disease |
| TCGA-H6-8124 | -1.977052415 | Chemotherapy | Complete Response |
